# BACE1^-/- ^mice exhibit seizure activity that does not correlate with sodium channel level or axonal localization

**DOI:** 10.1186/1750-1326-5-31

**Published:** 2010-08-23

**Authors:** Brian D Hitt, Thomas C Jaramillo, Dane M Chetkovich, Robert Vassar

**Affiliations:** 1 Department of Cell and Molecular Biology, Feinberg School of Medicine, Northwestern University, Chicago, IL 60611, USA; 2 Davee Department of Neurology and Clinical Neurosciences, Feinberg School of Medicine, Northwestern University, Chicago, IL 60611, USA; 3 Department of Physiology, Feinberg School of Medicine, Northwestern University, Chicago, IL 60611, USA

## Abstract

**Background:**

BACE1 is a key enzyme in the generation of the Aβ peptide that plays a central role in the pathogenesis of Alzheimer's disease. While BACE1 is an attractive therapeutic target, its normal physiological function remains largely unknown. Examination of BACE1^-/- ^mice can provide insight into this function and also help anticipate consequences of BACE1 inhibition. Here we report a seizure-susceptibility phenotype that we have identified and characterized in BACE1^-/- ^mice.

**Results:**

We find that electroencephalographic recordings reveal epileptiform abnormalities in some BACE1^-/- ^mice, occasionally including generalized tonic-clonic and absence seizures. In addition, we find that kainic acid injection induces seizures of greater severity in BACE1^-/- ^mice relative to BACE1^+/+ ^littermates, and causes excitotoxic cell death in a subset of BACE1^-/- ^mice. This hyperexcitability phenotype is variable and appears to be manifest in approximately 30% of BACE1^-/- ^mice. Finally, examination of the expression and localization of the voltage-gated sodium channel α-subunit Na_v_1.2 reveals no correlation with BACE1 genotype or any measure of seizure susceptibility.

**Conclusions:**

Our data indicate that BACE1 deficiency predisposes mice to spontaneous and pharmacologically-induced seizure activity. This finding has implications for the development of safe therapeutic strategies for reducing Aβ levels in Alzheimer's disease. Further, we demonstrate that altered sodium channel expression and axonal localization are insufficient to account for the observed effect, warranting investigation of alternative mechanisms.

## Background

Alzheimer's disease (AD) is a common and devastating neurodegenerative disorder involving a decline in memory and other cognitive functions. Disease modifying therapies for AD are greatly needed, but remain elusive. One promising approach to such a therapy is to inhibit the production of the β-amyloid (Aβ) peptide, which is the primary constituent of amyloid plaques that represent a major histopathological hallmark of AD [1,2]. Mutations that cause autosomal dominant familial AD (FAD) all lead to increased production of Aβ, particularly in its 42-amino acid isoform (Aβ_42_) (reviewed in [3]). This and other lines of evidence strongly suggest that Aβ plays a central and early role in AD pathogenesis (reviewed in [4]).

Aβ is produced through the endoproteolysis of the amyloid precursor protein (APP) by two proteases, the β- and γ-secretases (reviewed in [5]). APP is first cleaved by the β-secretase at the N-terminus of Aβ to produce the membrane-bound C99 fragment, which is further cleaved by γ-secretase to release Aβ. The β-secretase has been identified as a transmembrane aspartic protease referred to as BACE1 [6-10]. Because of its role in Aβ production, BACE1 is a promising drug target for AD. This is highlighted by the finding that Aβ generation, amyloid pathology, electrophysiological dysfunction, and cognitive deficits characteristic of APP transgenic mice are all abrogated by genetic deletion of BACE1 [11-15].

The normal function of BACE1 remains largely unknown, and a better understanding of its function(s) will be of value in anticipating potential adverse effects of BACE1 inhibition as a therapeutic strategy. In addition to APP, several other BACE1 substrates have been identified which may mediate the normal function of BACE1. These include α2,6-sialyltransferase [16], P-selectin glycoprotein ligand-1 (PSLG-1) [17], the APP homolog proteins APLP1 and APLP2 [18-20], low-density lipoprotein receptor-related protein (LRP) [21], the voltage-gated sodium channel β2 subunit (Na_v_β2) [22,23], neuregulin-1 (NRG1) [24,25] and neuregulin 3 (NRG3) [26]. We can also infer normal functions of BACE1 from deficits observed in the BACE1^-/- ^mouse lines that have been generated [11,27-29]. For instance, impaired performance in certain memory tasks suggests that BACE1 may play a role in memory [12,13]. In addition, reduced cleavage of NRG1 in BACE1^-/- ^mice has been shown to lead to hypomyelination in the central and peripheral nervous systems, as well as impaired remyelination following nerve injury [24-26]. This abrogated cleavage of NRG1, which is genetically linked to schizophrenia, has also been implicated in schizophrenia-like phenotypes described in BACE1^-/- ^mice [30].

BACE1 may, via its cleavage of Na_v_β2, affect the expression and function of voltage-gated sodium channels (VGSCs) and thus modulate membrane excitability. VGSCs are composed of a single pore-forming α-subunit and either one or two accessory β-subunits (reviewed in [31]). The β-subunits interact directly with the α-subunits to affect localization, cell-surface expression and inactivation of the VGSC [32] (reviewed in [31,33]). There are four β-subunits (β1-4), all of which appear to be cleaved by BACE1 [22,23]. Ten α subunits are known, four of which are notably found in the CNS: Na_v_1.1 and Na_v_1.3 in the neuronal soma and dendrite, and Na_v_1.2 and Na_v_1.6 in the axon (reviewed in [34]). BACE1 cleavage of β2 has been reported to increase expression of Na_v_1.1 *in vitro *and *in vivo*, though cell surface expression is reduced as the channel is retained intracellularly [35]. Interestingly, another study found that BACE1 alters sodium channel gating, leading to increased excitability, in a manner independent of proteolytic activity [36].

We have previously reported an increased sensitivity of BACE1^-/- ^mice to kainic acid-induced seizures [37]. Here we further characterize the seizure-susceptibility phenotype we have observed in BACE1^-/- ^mice. We report that a subset of these mice demonstrate abnormal background activity and spiking on EEG recording and a smaller subset display spontaneous tonic-clonic seizures. In addition, we find some BACE1^-/- ^mice to be particularly susceptible to pharmacologically induced seizures and excitotoxic injury. The previously reported regulation of VGSCs by BACE1 is an interesting candidate for an underlying mechanism of this phenotype, since mutations in both α and β-subunits have been linked to epilepsy [38-43] (reviewed in [44]). However, we find that neither total brain nor hippocampal protein nor axonal expression levels of sodium channels correlate with measures of seizure susceptibility in BACE1^-/- ^mice. Overall, our results suggest that BACE1 deficiency predisposes mice to neuronal hyperexcitability and that alterations of VGSC expression and axonal localization are insufficient to account for this effect.

## Results

### BACE1^-/- ^mice have spontaneous seizures and other EEG abnormalities

**Figure 1:**
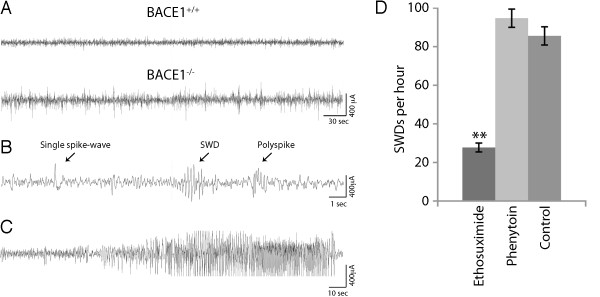
**Electoencephalogram (EEG) activity in BACE1^+/+ ^and BACE1^-/- ^mice**. **A**. Background spontaneous EEG activity in a BACE1^+/+ ^and BACE1^-/- ^mouse. **B**. Background EEG activity in BACE1^-/- ^mouse displays multiple epileptiform abnormalities. SWD, spike-wave discharge. **C**. EEG activity during a generalized tonic-clonic seizure in a BACE1^-/- ^mouse. **D**. Number of spike-wave discharges (SWDs) per hour observed in BACE1^-/- ^mice following administration of 100 mg/kg of ethosuximide or 25 mg/kg of phenytoin; n = 4; **p < 0.01.

In the course of maintaining the BACE1^-/- ^mice, we observed rare instances of spontaneous seizure-like behavior. To document and characterize these events, we recorded simultaneous EEG/video in BACE1^-/- ^mice at 3 months of age. A total of 16 BACE1^-/- ^mice were recorded continuously for five days. Whereas 11 of the 16 BACE1^-/- ^mice exhibited no epileptiform abnormalities or behavioral seizures, background EEG recordings in 5 of the 16 BACE1^-/- ^mice (Fig. 1A) revealed a heterogeneous mixture of epileptiform abnormalities that included single spike and polyspike abnormalities, as well as spike-wave discharges (SWD) (Fig. 1B) similar to those reported in mouse models of generalized absence epilepsy [45,46]. SWDs were observed in 4 of the 5 BACE1^-/- ^mice with abnormal EEG activity and occurred on average 85.6+3.4 events/hr during awake behavior, with spike frequency during discharges 5.8+1.2 Hz. Many, but not all of the SWDs observed on EEG were associated with distinct behavioural pauses, similar to findings in other models of generalized absence epilepsy [46]. Of the 5 BACE1^-/- ^mice exhibiting epileptiform background activity, 2 were observed to have electrographic and behavioural generalized tonic-clonic seizures (Fig. 1C; additional file 1: Video 1). Interestingly, all of the generalized tonic-clonic seizures in BACE1^-/- ^mice began during non-REM sleep, and were characterized by an abrupt loss of muscle tone (atonia) lasting 2-3 seconds (additional file 2: Video 2), followed immediately by tonic-clonic convulsions lasting from 1-4 minutes. Electrographically, seizures were characterized by an abrupt suppression of background EEG amplitude coincident with atonia, followed by continuous, large amplitude 6-8 Hz spike-wave activity that was accompanied by generalized convulsion. After convulsions, BACE1^-/- ^mice showed decreased movement and attenuated EEG amplitude lasting 1-2 minutes.

SWDs and absence seizures in mice and human patients can be reduced or prevented by treatment with the T-type Ca^2+^-channel antagonist, ethosuximide [46-48], whereas sodium channel antagonists such as phenytoin are not effective for treating absence seizures. Pharmacological studies in BACE1^-/- ^mice exhibiting SWDs revealed that whereas phenytoin had no effect on spike-wave discharges, ethosuximide markedly reduced the frequency of these discharges (Fig. 1D). Thus behavioural and electrographic findings, together with the observation of ethosuximide sensitivity of SWDs strongly suggest that BACE1^-/- ^mice have, in addition to generalized tonic-clonic seizures, absence seizures similar to those observed in other rodent models and human patients.

### Increased severity of KA-induced seizures in BACE1^-/- ^mice

**Figure 2:**
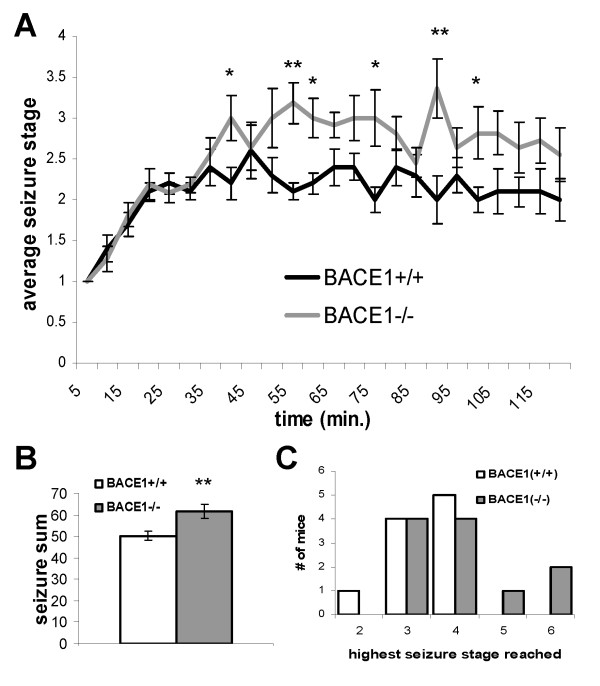
**BACE1^-/- ^mice have increased sensitivity to KA-induced seizures compared to wild-type littermates**. BACE1^-/- ^mice and wild-type littermate controls were injected with 15 mg/kg of KA and observed for 120 minutes. Seizures were rated (0-6) on a modified Racine scale at each 5-minute interval. **A**. Average seizure stage for each group over time. **B**. Sum of all seizure scores over 120 minutes. Error bars indicate SEM, * p < 0.05, **p < 0.01; n = 10, 11 for BACE1^+/+ ^and BACE1^-/- ^groups, respectively. **C**. Histogram of highest seizure stage reached overall by each mouse.

The finding that a fraction of BACE1^-/- ^mice have spontaneous seizures led us to consider whether pharmacological induction of seizures might produce different effects in BACE1^-/- ^and BACE1^+/+ ^mice. Kainic acid (KA) injection is used as an animal model of epileptic seizures [49]. Injected mice progress predictably through seizure stages of increasing severity. To test the hypothesis that BACE1^-/- ^mice have an increased susceptibility to KA-induced seizures, we injected 3-month-old BACE1^-/- ^mice and wild-type littermate controls intraperitoneally with 15 mg/kg of KA and observed their behaviour for 2 hours. This dose was determined by pilot studies to induce seizures of intermediate severity in wild-type mice. Seizures were rated by an observer blinded to genotype according to a modified version of the Racine scale, varying from 0 (no seizure) to 6 (severe tonic-clonic seizure)[49]. For each 5-minute interval post-injection, the highest seizure stage reached was recorded for each mouse. Beginning at 40 minutes post-injection, the average seizure stage of BACE1^-/- ^mice was greater than that of littermate controls, significantly so at many time points (Fig. 2A). A seizure sum was obtained for each mouse by adding the seizure scores from all 5-minute intervals of the 120-minute observation period. The average seizure sum of the BACE1^-/- ^mice was significantly higher than that of littermate controls (Fig. 2B; 61.6 ± 3.3 vs. 50.2 ± 2.2, p < 0.01, n = 11, 10 respectively).

At the dose of KA used (15 mg/kg), no wild-type mice had seizures more severe than stage 4. While most BACE1^-/- ^mice also reached seizure stages no higher than stage 4, 3 of 11 mice had more severe seizures (Figure 2C). These may represent a subset of BACE1^-/- ^mice that are particularly susceptible to excitotoxic seizures.

### BACE1^-/- ^mice exhibit KA-induced neurodegeneration

**Figure 3:**
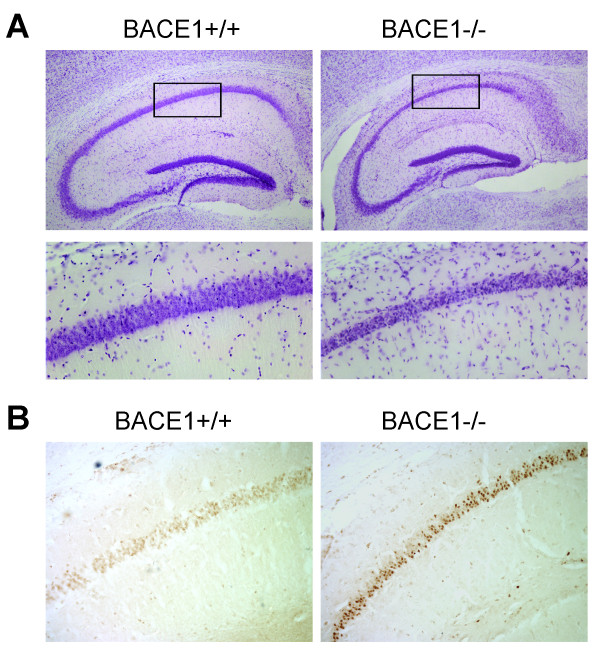
**KA-induced cell death in BACE1^-/- ^mice**. Mice were injected with 20 mg/kg of KA and recovered for 7 days before brains were harvested and sectioned. **A**. Cresyl violet staining reveals extensive degeneration in the CA1/2 region of the hippocampus in some BACE1^-/- ^mice (right) but no BACE1^+/+ ^littermates (left). 3 of 10 BACE1^-/- ^mice had cell loss as demonstrated. 0 of 7 wild-type mice had cell loss. Lower panels are higher magnification images of region indicated by box in upper panels. **B**. TUNEL staining performed on adjacent sections indicates DNA fragmentation in many cells of the CA1/2 region in 3 of 10 BACE1^-/- ^mice (right). No TUNEL-positive cells were seen in any of 7 KA-treated wild-type littermates (left) nor in BACE1^-/- ^mice that lacked cell loss.

A second cohort of mice was injected with a higher dose of KA (20 mg/kg) to assess the effect of BACE1 genetic deletion on excitotoxic cell death. All mice had severe (stage 5-6) seizures with this dose and mortality was ~15%. Brains from these mice were harvested after a 7-day recovery period. We stained brain sections with Cresyl violet in order to assess cell loss. The 20 mg/kg dose did not induce frank neurodegeneration in the hippocampi of any wild-type mice (n = 7). In contrast, a subset of the BACE1^-/- ^mice exposed to the high dose of KA demonstrated obvious cell loss in the CA1/2 region of the hippocampus (Fig. 3A). This cell loss occurred in 3 of the 10 BACE1^-/- ^mice that survived the 7-day recovery period. In these three brains, there was a notable thinning of the CA1/2 cell layer and nearly all nuclei appeared pyknotic throughout the CA1/2 region. The remaining BACE1^-/- ^mice and KA-treated BACE1^+/+ ^littermates did not demonstrate any apparent cell loss relative to saline-treated controls.

Since KA-induced cell death has been found to involve DNA fragmentation [50], we performed TUNEL staining on brain sections as a potentially more sensitive indicator of cell death. Robust TUNEL labelling was seen in the CA1/2 region of the hippocampus only in the mice that showed cell loss by Cresyl violet staining (Fig. 3B, right). No TUNEL-positive nuclei were observed in any other region of the hippocampus or the cortex in these mice. A fourth BACE1^-/- ^mouse had a small number of TUNEL-positive nuclei in the CA3 region (not shown). No TUNEL- positive nuclei were observed in BACE1^-/- ^mice that did not have cell loss (not shown) nor in any of the KA-treated wild-type littermate control mice (Fig. 3B, left; n = 7).

### Sodium channel protein levels are unaltered in BACE1^-/- ^mice

**Figure 4:**
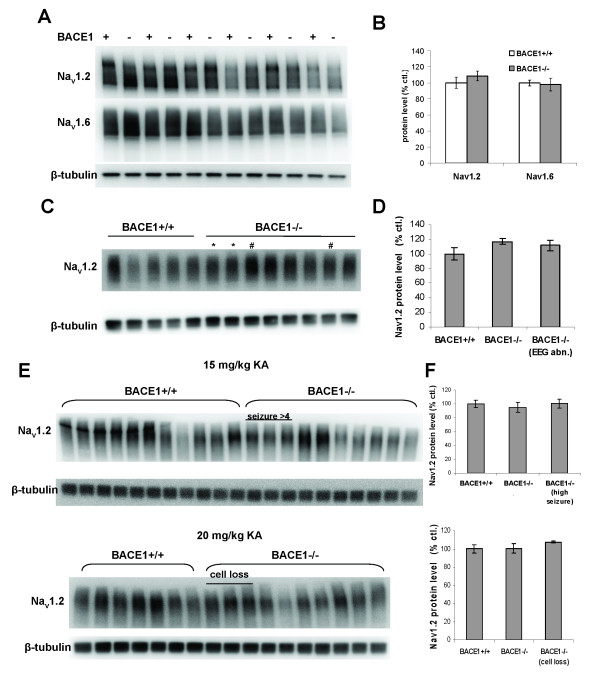
**No change in sodium channel total protein levels in BACE1^-/- ^mouse brain**. Whole brain or hippocampal homogenates from BACE1^-/- ^mice and wild-type littermate controls were resolved by SDS-PAGE for Western analysis with antibodies against Na_v_1.2 and Na_v_1.6. Samples in **A **and **B **are whole brain homogenates from naïve animals; Samples in **C **and **D **are hippocampal homogenates from mice subjected to EEG recordings; Samples in **E **and **F **are whole brain homogenates from mice injected with 15 mg/kg (top) or 20 mg/kg (bottom) KA. Panels **B**, **D **and **F **are quantifications of sodium channel signals performed on the blots in panels **A**, **C **and **E**, respectively. No significant differences were detected in any comparison. All measures were normalized using β-tubulin signal. Error bars indicate SEM. In **C**, * indicates mice that exhibited spontaneous seizures and # indicates mice that exhibited abnormal background/spiking on EEG. In **E**, mice with seizure >4 (high seizure) and cell loss are indicated.

The finding that BACE1 cleaves the β-subunits of voltage-gated sodium channels suggested a potential mechanism by which BACE1 might affect neuronal excitability [22,23]. To determine whether altered levels of voltage-gated sodium channels in the brain are responsible for the hyperexcitability of BACE1^-/- ^mice, we performed Western blot analysis on brain homogenates from BACE1^-/- ^mice and BACE1^+/+ ^littermates. Given the apparent presynaptic localization of BACE1 [13,51], we specifically examined Na_v_1.2, an alpha subunit with axonal distribution in the CNS [34]. A Western blot of whole brain homogenates shows similar levels of Na_v_1.2 as well as Na_v_1.6, the other prominent axonal CNS α-subunit, in the brains of BACE1^-/- ^mice and wild-type littermate controls (Fig. 4A). Quantification showed no significant difference in either case (Na_v_1.2: 100 ± 7.0 (+/+) vs 108.7 ± 6.1 (-/-), Na_v_1.6: 100 ± 3.7 (+/+) vs 98.0 ± 7.9 (-/-), n = 6).

Our characterization of the seizure phenotype demonstrated a large variability within the group of BACE1^-/- ^mice in their susceptibility to seizure/excitotoxicity. We therefore tested the hypothesis that the subset of BACE1^-/- ^mice that appear particularly susceptible have an increase in brain sodium channel levels that is not apparent in the group as a whole. To this end we first compared hippocampal homogenates from the brains of mice we had characterized with EEG recordings (Fig. 4C,D). These included 2 mice that demonstrated spontaneous seizures (*) and 2 mice with abnormal background/spiking (#), as well as 4 with normal EEGs. No significant difference in Na_v_1.2 levels in BACE1^-/- ^hippocampi compared with wild-type littermate controls was observed, either in those with abnormal EEGs or in the group as a whole (Fig. 4D) (+/+: 100 ± 8.3, n = 5, -/-: 116.9 ± 4.1, n = 8, EEG abn.: 111.5 ± 7.2, n = 4).

We next compared brain homogenates from the cohorts of mice treated with 15 mg/kg and 20 mg/kg KA (Fig 4E). The low dose cohort included a subset of 3 mice that reached seizure stages >4, while the high dose cohort included the 3 mice that demonstrated frank cell loss in the hippocampus. In both 15 mg/kg and 20 mg/kg KA groups, no differences in Na_v_1.2 levels were seen in either the BACE1^-/- ^group or the subgroups that showed increased susceptibility (Fig. 4F) (+/+: 100 ± 5.4, -/-: 94.9 ± 7.3, -/- seiz. > 4: 100.5 ± 6.6, n = 11, 10, 3 respectively; +/+: 100 ± 4.8, -/-: 100.5 ± 5.2, -/- cell loss: 107.7 ± 0.9, n = 7, 10, 3 respectively). While our results demonstrate a large degree of variability in protein levels of voltage-gated sodium channels in mouse brain, such levels do not correlate with BACE1 genotype nor seizure phenotype.

### Na_v_1.2 levels in mossy fibers do not correlate with seizure phenotype

**Figure 5:**
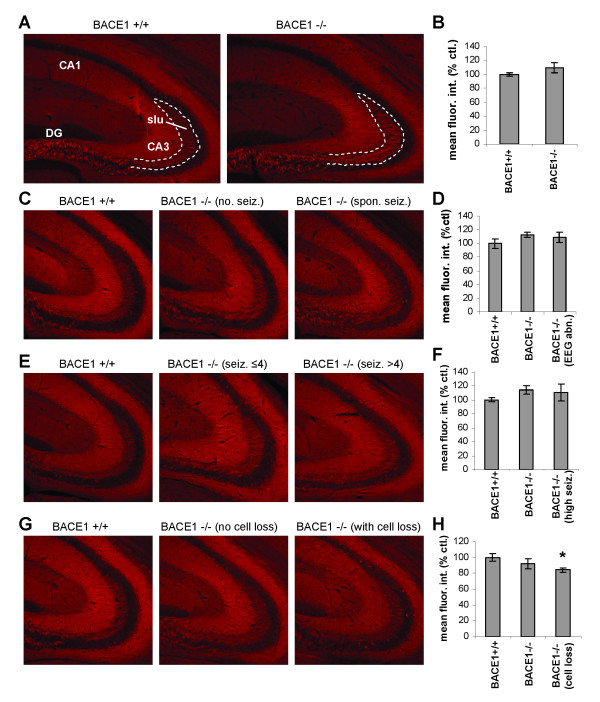
**Na_V_1.2 levels in mossy fibers do not correlate with seizure measures**. Immunofluorescence labelling of Na_v_1.2 was performed on 30 μm brain sections, and confocal images of the CA3 region of the hippocampus were obtained. **slu**, stratum lucidum; **DG**, dentate gyrus. Sections in **A **are representative images from naïve mice, **C**, from mice subjected to EEG recordings, **E**, from mice treated with 15 mg/kg KA, and **G**, from mice treated with 20 mg/kg KA. Panels **B**, **D**, **F **and **H **are histograms representing quantifications of Na_v_1.2 fluorescence intensity in the stratum lucidum measured by ImageJ of all mice from the groups indicated. Mean fluorescence intensity within the stratum lucidum was measured and averaged over 2-4 sections/brain. In **D**, **F**, and **H **the subgroups with EEG abnormalities (EEG abn.) and severe seizures (high seiz.) did not have higher axonal Na_v_1.2 levels than the BACE1^-/- ^group as a whole (middle bars). BACE1^-/- ^mice with cell loss following 20 mg/kg KA treatment had significantly reduced Na_v_1.2 in the stratum lucidum. Error bars indicate SEM. n = 3 brains in **B**; n = 5, 13, 5 resp. in **D**; n = 3, 6, 3 resp. in **F**; n = 3, 6, 3 resp. in **H**. * p < 0.05. (no seiz., mice with no seizures; spon. seiz., mice with spontaneous seizures; seiz. ≤4, mice with seizures less than or equal to stage 4; seiz. >4, mice with seizures greater than stage 4)

Some reports suggest that BACE1 may have different or even opposite effects on sodium channel expression levels versus cell surface localization [35,52]. Although we found total protein levels of Na_v_1.2 to be unchanged in BACE1^-/- ^brains, we considered that altered sodium channel density on the surface of axons might underlie the hyperexcitability phenotype. To initially investigate this, we labelled Na_v_1.2 in coronal brain sections of naïve mice using fluorescent antibodies and analyzed confocal images of the hippocampus. We examined the stratum lucidum in CA3 (Fig. 5A, slu), which contains the axons of the mossy fiber pathway, since robust BACE1 expression is seen in the mossy fiber pathway [13,51]. Quantitative analysis of fluorescence intensity in the stratum lucidum indicated that there is no significant difference between Na_v_1.2 levels in BACE1^-/- ^brains relative to wild-type littermates (Fig. 5B; +/+: 100 ± 2.0, -/-: 109.9 ± 7.0, n = 3).

To determine whether differences in axonal sodium channel levels might account for the variability of seizure outcomes, the subgroups with abnormal EEGs, severe seizures, and KA-induced cell loss were analyzed separately. No significant difference was detected between Na_v_1.2 levels in the stratum lucidum of BACE1^-/- ^brains versus BACE1^+/+ ^brains, nor did those mice that had spontaneous seizures and/or abnormal spiking on EEG have higher Na_v_1.2 levels than the BACE1^-/- ^group as a whole (Fig. 5C, D) (+/+: 100 ± 6.6, n = 5; -/-: 112.2 ± 3.7, n = 13; EEG abn.: 109.2 ± 7.8, n = 5). Similarly, the BACE1^-/- ^mice that reached seizure stages >4 when treated with 15 mg/kg KA did not have greater stratum lucidum Na_v_1.2 levels than other BACE1^-/- ^mice, and the BACE1^-/- ^group did not vary significantly from wild-type mice (Fig. 5E, F) (+/+: 100 ± 3.0, n = 3; -/-: 114 ± 5.7, n = 6; seiz. > 4: 110.6 ± 11.8, n = 3). On the other hand, BACE1^-/- ^mice that incurred cell loss following treatment with 20 mg/kg KA had significantly less Na_v_1.2 staining in stratum lucidum than wild-type littermates (Fig. 5G, H) (+/+: 100 ± 4.5, n = 3; -/-: 92.2 ± 6.0, n = 6; cell loss: 84.5 ± 2.6, p = 0.04 rel. to +/+, n = 3). This is most likely due to KA-induced axonal degeneration.

## Discussion

While the role of BACE1 in the production of Aβ makes it a key enzyme in AD pathogenesis, little is known about its normal physiological function. A better understanding of the consequences of BACE1 deficiency will aid in the design of therapeutic strategies to abrogate Aβ generation with minimal adverse effects. Our results demonstrate that genetic deletion of BACE1 in mice leads to an increased susceptibility to spontaneous and pharmacologically-induced seizures. The observation of rare spontaneous tonic-clonic seizures in BACE1^-/- ^mice prompted us to monitor a cohort of these mice with simultaneous EEG/video recordings. Our data indicate that a subset of BACE1^-/- ^mice are epileptic, with spontaneous epileptiform abnormalities occurring in ~30% of animals studied by video and EEG. Of the animals with epileptiform abnormalities, 80% exhibited spike-wave discharges and behavioural pauses consistent with absence seizures, whereas 40% were observed to have generalized tonic-clonic convulsions. Because our recording was limited to 5 days of continuous recording, it is possible that a higher proportion of animals with abnormal EEGs could also have spontaneous generalized tonic-clonic seizures. Longer recording periods and evaluation of age-dependent changes in seizure patterns are planned for future studies that will allow better characterization of the seizure phenotypes present in these animals.

The anatomical basis for epilepsy in the BACE1^-/- ^mice is unclear. SWDs and absence seizures are thought to represent aberrant function in the circuitry that reciprocally connects the thalamus and cortex (for review, see [53]). Along these lines, abnormal excitability of thalamocortical neurons, cortical pyramidal neurons, and intrathalamic inhibitory neurons have each been suggested to contribute to genesis of seizures in models of absence epilepsy. At the molecular level abnormal function of T-type voltage gated calcium channels, hyperpolarization-activated cyclic nucleotide-gated channels, voltage gated chloride channels as well as abnormalities of glutamatergic and GABAergic neurotransmission have been implicated as playing a role in absence seizures. Apart from absence seizures, BACE1^-/- ^mice also demonstrate more severe seizures and hippocampal injury following administration of KA, suggesting that abnormal excitability is not restricted to neurons involved in thalamocortical pathways. Furthermore, BACE1^-/- ^mice also exhibit generalized tonic-clonic seizures. Understanding whether these convulsive seizures in BACE1^-/- ^mice are a result of generalized neuronal hyperexcitability leading to generalized synchronous brain activity at seizure onset, or begin with abnormal synchrony in one or more focal regions of brain, is a goal of future research.

The fact that electrographic and video evidence of spontaneous tonic-clonic seizures was only obtained for 2 of the 16 BACE1^-/- ^mice that we monitored indicates a low penetrance of the seizure phenotype in the BACE1^-/- ^population. Additional BACE1^-/- ^mice displayed other abnormal findings on EEG such as single spikes and polyspike complexes; however these still represent a minority of BACE1^-/- ^mice (5 of 16, ~31%). This incomplete penetrance suggests that while BACE1-deficiency contributes to seizure susceptibility, mediating factors exist that have yet to be determined. Given that the BACE1^-/- ^mice are maintained on an inbred C57/BL6 background, such factors are likely to be developmental/environmental.

The results of our studies using mice treated with the glutamate analogue kainic acid (KA) reveal a similar incompletely-penetrant phenotype in the BACE1^-/- ^mice. BACE1^-/- ^mice treated with 15 mg/kg of KA had significantly higher ratings of seizure severity on average than BACE1^+/+ ^littermates. However, the highest seizure stage reached by most BACE1^-/- ^mice was stage 3 or 4, as seen in wild-type mice. A subset of 3 of 11 (~27%) BACE1^-/- ^mice reached seizure stage 5 or 6, which is atypical at this dose of KA. This again suggests that a fraction of BACE1^-/- ^mice have a particularly marked increase in their susceptibility to seizures. Similarly, 3 of 10 (30%) BACE1^-/- ^mice injected with 20 mg/kg of KA displayed extensive excitotoxic cell death in the CA1/2 region of the hippocampus. No cell loss was seen in the remaining BACE1^-/- ^mice nor in any of the treated BACE1^+/+ ^littermates. While it is not clear whether the subgroups from these three experiments represent the same population, the finding of an incomplete penetrance of approximately 30% is consistent across different measures of susceptibility to seizure and excitotoxicity, highlighting the existence of additional factors mediating the expression of this phenotype in BACE1^-/- ^mice.

The cleavage by BACE1 of the β-subunits of VGSCs has been purported to alter the expression and localization of VGSC α-subunits [35], effects that may bear on the hyperexcitability phenotype discussed here. We addressed this possibility by examining Na_v_1.2 expression levels in the brain and localization to the axons of the mossy fiber pathway in the hippocampus. We chose this approach because of the high concentration of BACE1 in the mossy fiber presynaptic terminals [13,51] and the particular susceptibility of hippocampal neurons to excitotoxicity in seizure models [50]. Our results indicate that brain levels of Na_v_1.2 protein are no different in BACE1^-/- ^mice relative to BACE1^+/+ ^mice, though there was a large degree of variability between mice. In addition, protein levels of Na_v_1.2 from the brains of mice identified by our EEG and KA-injection experiments as particularly susceptible to seizure and excitotoxic injury did not differ from those of BACE1^+/+ ^mice or BACE1^-/- ^mice as a whole. Similar results were obtained from our measurements of the intensity of immunofluorescent labelling of Na_v_1.2 in the stratum lucidum (the location of mossy fiber axons). There was no statistically significant difference between Na_v_1.2 staining in the mossy fibers of BACE1^-/- ^mice and those of BACE1^+/+ ^mice, and axonal Na_v_1.2 levels were not higher in mice with EEG abnormalities or severe KA-induced seizures than in BACE1^-/- ^mice as a whole. Thus, while there is large variation across the BACE1^-/- ^mouse population in seizure susceptibility as well as in Na_v_1.2 expression and axonal localization, our results find no correlation of these measures and do not support the hypothesis that altered Na_v_1.2 expression or localization underlies the hyperexcitability phenotype of BACE1^-/- ^mice.

While we were preparing this manuscript, a study by Hu *et al. *was published that reports a similar seizure phenotype in BACE1^-/- ^mice and also examines VGSC expression and function [52]. Hu *et al. *report that the fraction of BACE1^-/- ^mice that develop spontaneous seizures detected by EEG monitoring increases with age, reaching 21.9% by age >10 months, and find that BACE1^-/- ^mice injected with KA tend to reach higher seizure stages than BACE1^+/+ ^mice [52]. They present compelling evidence of increased neuronal excitability in BACE1^-/- ^brain slices using extracellular field recordings and of increased sodium currents and altered firing properties in dissociated BACE1^-/- ^neurons using patch-clamp and current-clamp recordings. In addition, they report reduced total Na_v_1.2 protein levels and increased cell surface expression and mossy fiber localization of Na_v_1.2 in BACE1^-/- ^brains, consistent with the findings of Kim *et al. *for Na_v_1.1 [35]. The characteristics of the seizure susceptibility phenotype of BACE1^-/- ^mice that we present here are quite consistent with those reported by Hu *et al. *However, we do not find a reduction in the total protein levels of VGSC α-subunits (Na_v_1.2 or Na_v_1.6) in BACE1^-/- ^brains, as reported by Hu *et al. *[52]. The cause of this discrepancy is not clear but may be related to differing extraction methods or the more robust sample size in our experiments given the high variability of sodium channel levels between animals. Hu *et al. *report a qualitative increase in Na_v_1.2 immunofluorescent labelling in the stratum lucidum but do not quantify this increase and do not address the implied causal link to the seizure/neuronal excitability phenotype experimentally. Our quantification of Na_v_1.2 immunofluorescence intensity in the strata lucida of BACE1^-/- ^and BACE1^+/+ ^brain sections shows no significant difference and finds that these levels do not correlate with EEG abnormalities or severity of KA-induced seizures as one would expect if increased Na_v_1.2 expression in axons were responsible for these outcomes.

Our results support the conclusion that VGSC level and localization in BACE1^-/- ^mice are insufficient to account for the predisposition to seizures in these mice. Thus, further investigation of other potential contributing mechanisms is warranted. One such mechanism is the effect of BACE1 on sodium channel gating properties as reported by Huth *et al. *[36]. This phenotype may also be due to the effects of the proteolysis of other proteins identified as BACE1 substrates, such as α2,6-sialyltransferase, PSLG-1, APLP1, APLP2, LRP, NRG1 and NRG3, or currently undiscovered BACE1 substrates. The hypomyelination purportedly found in the CNS of BACE1^-/- ^mice [24] may also contribute. Alternatively, the seizure susceptibility of BACE1^-/- ^mice may be secondary to hypothetically altered formation of synapses and neural circuits during brain development. There is robust expression of BACE1 in the brain during early postnatal development [25,54], during which time, proteolysis of BACE1 substrates may play a role in synapse formation and patterning. Finally, the effect of BACE1 on neuronal excitability may involve the action of Aβ at synapses. It has been observed that Aβ is released at synapses in an activity-dependent manner [55] and that Aβ can negatively regulate excitatory neurotransmission via reduction of AMPA receptors [56,57]. These processes should not be discounted as potential contributors to the hyperexcitability phenotype observed in BACE1^-/- ^mice.

## Conclusions

We have demonstrated that a subset of BACE1^-/- ^mice displays abnormal brain activity on EEG, occasionally including spontaneous absence and tonic-clonic seizures. In addition, BACE1^-/- ^mice have KA-induced seizures of greater severity than BACE1^+/+ ^littermates, and a fraction of KA-treated mice exhibit excitotoxic cell death in the hippocampus that is not observed in wild-type mice. While the regulation of VGSCs by BACE1 may contribute to this seizure-susceptibility phenotype, we find that neither Na_v_1.2 expression levels nor axonal localization correlate with seizure susceptibility, indicating that alteration of these properties is insufficient to fully explain the phenotype. Thus, research into other potential mechanisms is warranted, as is research into factors that mediate the expression of the phenotype given its incomplete penetrance. Better understanding of the effect of BACE1 deficiency on neuronal excitability and seizure susceptibility has important implications for BACE1 inhibition as a therapeutic strategy for AD.

## Methods

### Animals

BACE1^-/- ^mice were purchased from The Jackson Laboratory, Bar Harbor, ME, USA (Described in [27]). Heterozygotes were used for breeding and all litters were genotyped by PCR using primers listed in the supplementary information of [27]. All wild-type controls used were BACE1^+/+ ^littermates. All mice were maintained in microisolator cages in the Barrier Facilities of Northwestern University Center for Comparative Medicine. All animal procedures were in strict accordance with the National Institutes of Health *Guide for the Care and Use of Laboratory Animals *and were approved by the Northwestern University Animal Care and Use Committee.

### Animal surgery, recording, and pharmacology

Animal surgery was performed as described in [46]. In summary, BACE1^-/- ^and BACE1^+/+ ^mice were anesthetized by intraperitoneal injection of xylazine/ketamine (100 mg/kg/10 mg/kg). The scalp was exposed and four holes were drilled to the dura: two placed 1 mm anterior to bregma, and two placed 7 mm anterior to bregma, each was 1.5 mm lateral to the central sulcus. A prefabricated headmount was attached to the skull with stainless steel screws and covered with dental acrylic. Following 1 week of recovery, a preamplifier was attached to the headmount and a data acquisition system (Pinnacle Technology Inc., Lawrence, KS) was used to record continuous EEG and video in freely moving mice. BACE1^-/- ^mice were placed in a recording chamber with food and water and allowed to freely move while recording. To screen for abnormal paroxysmal EEG activity BACE1^-/- ^mice were recorded continuously for five days. Following acquisition of baseline EEG activity, mice with abnormal EEG activity were used in pharmacology studies. BACE1^-/- ^mice displaying seizure activity received a single intraperitoneal injection of phenytoin (25 mg/kg; Sigma, St. Louis, MO) or ethosuximide (Sigma, 100 mg/kg). Spike wave discharges (SWDs) were scored for 1 hour following injection. SWDs were characterized as bilaterally synchronous, regular (4-6 Hz) multiple-spike complexes with a spike and wave morphology and amplitudes at least 4 times higher than average baseline amplitude [58]. Single spikes were identified as sharp activity with amplitude of at least 4 times baseline amplitude associated with an aftercoming slow wave. Polyspike complexes were identified as multiple independent spikes without associated wave morphology. EEG activity 1 hour post-injection was analyzed for SWDs as compared to 1-hour epochs preceding the injection in the same BACE1^-/- ^mice.

### Kainic acid treatment and seizure assessment

At three months of age, mice received intraperitoneal injections of either 15 mg/kg or 20 mg/kg kainic acid (Tocris, Bristol, UK, Cat. 0222) and were observed for two hours (15 mg/kg) or four hours (30 mg/kg). Seizure stage was assessed according to a modification of the Racine scale: stage 0 - normal behaviour; stage 1 - hypoactivity, immobility; stage 2 - rigidity, whisker twitching; stage 3 - reared, rigid posture, some automatisms (e.g. forelimb pawing, head bobbing, tail whipping); stage 4 - intermittent rearing and falling with forelimb/jaw clonus, stage 5 - continuous rearing and falling >30 s or continuous jumping (popcorning); stage 6 - generalized tonic-clonic seizures with whole body convulsions. For each five-minute interval the highest seizure stage reached was recorded.

### Histology and TUNEL staining

At seven days post-injection, mice were deeply anesthetized with intraperitoneal injection of ketamine (200 mg/kg)/xylazine (25 mg/kg) and then transcardially perfused with HEPES buffer containing protease inhibitors [20 μg/ml phenylmethylsulphonyl fluoride, 0.5 μg/ml leupeptin, 20 μM sodium orthovanadate, and 100 μM dithiothreitol (DTT)] before removal of brains. One hemibrain per mouse was fixed in 4% paraformaldehyde (PFA) in PBS for 24 h and then cryopreserved in 30% (w/v) sucrose for >24 h. 30 μm coronal sections were cut from PFA-fixed brains on a freezing sliding microtome and collected in 0.1 M PBS with 0.01% sodium azide. Sections were mounted on slides and stained with cresyl violet. TUNEL staining was performed on slide-mounted brain sections surrounded by ImmEdge hydrophobic barrier (Vector Laoratories, Burlingame, CA). Slides were washed in PBS + 0.1% Tween-20 (PBS-T) between all steps. Tissue was permeabilized by treatment with proteinase K (20 μg/ml) in PBS-T for 30 min., treated with 3% H_2_0_2 _in PBS for 10 min., and equilibrated in TdT reaction buffer for 10 min. before incubation in dUTP-biotin (5 μM) and terminal deoxynucleotidyl transferase (TdT) enzyme (2000 U/ml) (Roche Diagnostics, Mannheim, Germany) in a humidified chamber at 37°C for 2 hours. Reaction was stopped by placing slides in 300 mM NaCl, 30 mM Na Citrate for 10 min. Sections were then incubated in reagents A and B from Vectastain ABC kit (Vector Laoratories, Burlingame, CA) (10 μl each per ml PBS-T) for 30 min., washed, and incubated in a 1:1 mix of 0.67 ul/ml 30% H_2_O_2 _in H_2_O: 1 mg/ml 3,3-Diaminobenzidine (DAB) in PBS for 5 min.. A section treated with reaction mixture excluding TdT enzyme was used as a negative control and a section pretreated with DNaseI (3000 u/ml) (Roche Diagnostics, Mannheim, Germany) was used as a positive control.

### Immunoblotting

Flash-frozen hemibrains or hippocampi were homogenized in PBS 1% Triton-X 100 with 1× Calbiochem Protease Inhibitor Cocktail Set I (EMD Biosciences, La Jolla, CA) using a motorized tissue homogenizer (hemibrains) or a tube and pestle (hippocampi). Total protein concentrations of brain homogenates were determined by the BCA method (Pierce, Rockford, IL). 5 μg protein from brain homogenates was boiled in SDS sample boiling buffer before being separated on 4%-12% NuPAGE Bis-Tris gels in 1× MOPS running buffer (Invitrogen, Carlsbad, CA) and transferred to Millipore Immobilon-P polyvinylidene difluoride (PVDF) membrane (Millipore, Billerica, MA), as described previously [51]. Blots were blocked in 5% non-fat dry milk in Tris-buffered saline (TBS), 0.1% Tween 20 (TBS-T; Sigma) overnight, then incubated in primary antibody (Na_v_1.2: Rb pAb, Millipore, AB 5206, 1:1000; Na_v_1.6: Rb pAb, Abcam, Cambridge, MA, ab65166, 1:500; β-tubulin, Ms mAb, Millipore, MAB5564, 1:50000) for 1 hr at RT or overnight at 4°C. Blots were washed in TBST and incubated for 1 hr in horseradish peroxidase (HRP)-conjugated goat anti rabbit (Jackson ImmunoResearch Laboratories, West Grove, PA) or horse anti-mouse (Vector Laboratories) secondary antibodies diluted 1:10,000 in 5% milk in TBST. Immunosignals were detected using enhanced chemiluminescence (ECL or ECL+, Amersham Biosciences, Piscataway, NJ) and quantified using a Kodak Image Station 400R imager (Rochester, NY). Densitometric analyses of immunoblots were performed using Kodak Molecular Imaging Software SE. Immunosignals were normalized to β-tubulin immunosignal. Values were expressed as percentages of the mean of the control. Statistical significance was determined using unpaired two-tailed Student's t-test to compare each BACE1^-/- ^group (total or subset) with BACE1^+/+ ^controls and two-way ANOVA to compare three groups.

### Immunofluorescence labelling and confocal microscopy

Tissue sections were prepared as described above. Sections with equivalent rostral-caudal locations were selected using anatomical landmarks and the size and shape of the hippocampus and ventricles. 2 sections (naïve and KA-treated mice) or 4 sections (EEG mice) were selected per brain. Free-floating sections were washed in TBS + 0.25% Triton-X 100 and blocked for 90 min. in 5% goat serum before being incubated in primary antibody (Rb anti-Na_v_1.2 pAb, Millipore, AB5206, 1:500) in TBS + 0.25% Triton-X 100, 1% bovine serum albumin (BSA) at 4°C overnight on an orbital shaker. Sections were then washed in Triton-TBS, 1% BSA and incubated in 1:10,000 goat anti-rabbit Alexa Fluor 594 antibody (Invitrogen) in Triton-TBS, 1% BSA for 90 min. on an orbital shaker. Sections were washed in TBS and mounted on slides. Coverslips were applied with Prolong Gold anti-fade mounting media (Invitrogen). Confocal images were acquired using a Nikon C1Si confocal microscope (Tokyo, Japan) with a 10× air objective. Laser power percentage, gain, and offset settings were held constant for all images acquired and saturation was never reached. All images were acquired during one continuous session to prevent effects of decay of laser intensity. Images were analyzed using ImageJ (NIH). The stratum lucidum was outlined in each image using a freeform marquee and mean brightness was measured within the outlined area. Mean fluorescence intensity values for each brain were averages of sections from the same brain and means and SEMs were calculated for each treatment group. Values were expressed as percentages of the mean of the control. Statistical significance was determined using unpaired two-tailed Student's t-test to compare each BACE1^-/- ^group (total or subset) with BACE1^+/+ ^controls and two-way ANOVA to compare three groups.

## Competing interests

The authors declare that they have no competing interests.

## Authors' contributions

BH participated in the design of the study, carried out the kainic acid studies, histology, immunoblotting, and immunofluorescence/microscopy and drafted the manuscript. TJ and DC designed and carried out the video/electrographic recordings and pharmacological studies and helped to draft the manuscript. RV conceived of the study, and participated in its design and coordination and helped to draft the manuscript. All authors read and approved the final manuscript.

## Supplementary Material

Additional file 1 **Video 1. Video monitoring and corresponding EEG trace of BACE1-/- mouse exhibiting typical generalized tonic-clonic seizure**. Click here for file

Additional file 2 **Video 2. Video monitoring of the onset of 3 distinct generalized tonic-clonic seizures in BACE1^-/- ^mice, demonstrating atonia at seizure onset**. Click here for file

## References

[B1] Glenner GG Wong CW Alzheimer's disease and Down's syndrome: sharing of a unique cerebrovascular amyloid fibril protein Biochem Biophys Res Commun 1984 122 1131 1135 10.1016/0006-291X(84)91209-9 6236805

[B2] Masters CL Multhaup G Simms G Pottigiesser J Martins RN Beyreuther K Neuronal origin of a cerebral amyloid: neurofibrillary tangles of Alzheimer's disease contain the same protein as the amyloid of plaque cores and blood vessels EMBO Journal 1985 4 2757 2763 4065091 10.1002/j.1460-2075.1985.tb04000.x PMC554575

[B3] Sisodia SS St George-Hyslop PH gamma-Secretase, Notch, Abeta and Alzheimer's disease: where do the presenilins fit in? Nat Rev Neurosci 2002 3 281 290 10.1038/nrn785 11967558

[B4] Selkoe DJ Biochemistry and Molecular Biology of Amyloid beta-Protein and the Mechanism of Alzheimer's Disease Handb Clin Neurol 2008 89 245 260 full_text 18631749 10.1016/S0072-9752(07)01223-7

[B5] Tanzi RE Bertram L Twenty years of the Alzheimer's disease amyloid hypothesis: a genetic perspective Cell 2005 120 545 555 10.1016/j.cell.2005.02.008 15734686

[B6] Hussain I Powell D Howlett DR Tew DG Meek TD Chapman C Gloger IS Murphy KE Southan CD Ryan DM Smith TS Simmons DL Walsh FS Dingwall C Christie G Identification of a novel aspartic protease (Asp 2) as beta-secretase Molecular and Cellular Neuroscience 1999 14 419 427 10.1006/mcne.1999.0811 10656250

[B7] Sinha S Anderson JP Barbour R Basi GS Caccavello R Davis D Doan M Dovey HF Frigon N Hong J Jacobson-Croak K Jewett N Keim P Knops J Lieberburg I Power M Tan H Tatsuno G Tung J Schenk D Seubert P Suomensaari SM Wang S Walker D Zhao J McConlogue L John V Purification and cloning of amyloid precursor protein beta-secretase from human brain Nature 1999 402 537 540 10.1038/990114 10591214

[B8] Vassar R Bennett BD Babu-Khan S Kahn S Mendiaz EA Denis P Teplow DB Ross S Amarante P Loeloff R Luo Y Fisher S Fuller J Edenson S Lile J Jarosinski MA Biere AL Curran E Burgess T Louis JC Collins F Treanor J Rogers G Citron M Beta-Secretase cleavage of Alzheimer's amyloid precursor protein by the transmembrane aspartic protease BACE Science 1999 286 735 741 10.1126/science.286.5440.735 10531052

[B9] Yan R Bienkowski MJ Shuck ME Miao H Tory MC Pauley AM Brashler JR Stratman NC Mathews WR Buhl AE Carter DB Tomasselli AG Parodi LA Heinrikson RL Gurney ME Membrane-anchored aspartyl protease with Alzheimer's disease beta-secretase activity Nature 1999 402 533 537 10.1038/990107 10591213

[B10] Lin X Koelsch G Wu S Downs D Dashti A Tang J Human aspartic protease memapsin 2 cleaves the β-secretase site of β-amyloid precursor protein Proc Natl Acad Sci USA 2000 97 1456 1460 10.1073/pnas.97.4.1456 10677483 PMC26455

[B11] Luo Y Bolon B Kahn S Bennett BD Babu-Khan S Denis P Fan W Kha H Zhang J Gong Y Martin L Louis JC Yan Q Richards WG Citron M Vassar R Mice deficient in BACE1, the Alzheimer's beta-secretase, have normal phenotype and abolished beta-amyloid generation Nature Neurosci 2001 4 231 232 10.1038/85059 11224535

[B12] Ohno M Sametsky EA Younkin LH Oakley H Younkin SG Citron M Vassar R Disterhoft JF BACE1 Deficiency Rescues Memory Deficits and Cholinergic Dysfunction in a Mouse Model of Alzheimer's Disease Neuron 2004 41 27 33 10.1016/S0896-6273(03)00810-9 14715132

[B13] Laird FM Cai H Savonenko AV Farah MH He K Melnikova T Wen H Chiang HC Xu G Koliatsos VE Borchelt DR Price DL Lee HK Wong PC BACE1, a major determinant of selective vulnerability of the brain to amyloid-beta amyloidogenesis, is essential for cognitive, emotional, and synaptic functions J Neurosci 2005 25 11693 11709 10.1523/JNEUROSCI.2766-05.2005 16354928 PMC2564291

[B14] Ohno M Cole SL Yasvoina M Zhao J Citron M Berry R Disterhoft JF Vassar R BACE1 gene deletion prevents neuron loss and memory deficits in 5XFAD APP/PS1 transgenic mice Neurobiology of disease 2007 26 134 145 10.1016/j.nbd.2006.12.008 17258906 PMC1876698

[B15] McConlogue L Buttini M Anderson JP Brigham EF Chen KS Freedman SB Games D Johnson-Wood K Lee M Zeller M Liu W Motter R Sinha S Partial reduction of BACE1 has dramatic effects on Alzheimer plaque and synaptic pathology in APP Transgenic Mice The Journal of biological chemistry 2007 282 26326 26334 10.1074/jbc.M611687200 17616527

[B16] Kitazume S Tachida Y Oka R Shirotani K Saido TC Hashimoto Y Alzheimer's beta-secretase, beta-site amyloid precursor protein-cleaving enzyme, is responsible for cleavage secretion of a Golgi-resident sialyltransferase Proc Natl Acad Sci USA 2001 98 13554 13559 10.1073/pnas.241509198 11698669 PMC61079

[B17] Lichtenthaler SF Dominguez DI Westmeyer GG Reiss K Haass C Saftig P De Strooper B Seed B The cell adhesion protein P-selectin glycoprotein ligand-1 is a substrate for the aspartyl protease BACE1 The Journal of biological chemistry 2003 278 48713 48719 10.1074/jbc.M303861200 14507929

[B18] Eggert S Paliga K Soba P Evin G Masters CL Weidemann A Beyreuther K The proteolytic processing of the amyloid precursor protein gene family members APLP-1 and APLP-2 involves alpha-, beta-, gamma-, and epsilon-like cleavages: modulation of APLP-1 processing by n-glycosylation The Journal of biological chemistry 2004 279 18146 18156 10.1074/jbc.M311601200 14970212

[B19] Li Q Sudhof TC Cleavage of amyloid-beta precursor protein and amyloid-beta precursor-like protein by BACE 1 The Journal of biological chemistry 2004 279 10542 10550 10.1074/jbc.M310001200 14699153

[B20] Pastorino L Ikin AF Lamprianou S Vacaresse N Revelli JP Platt K Paganetti P Mathews PM Harroch S Buxbaum JD BACE (beta-secretase) modulates the processing of APLP2 in vivo Mol Cell Neurosci 2004 25 642 649 10.1016/j.mcn.2003.12.013 15080893

[B21] von Arnim CA Kinoshita A Peltan ID Tangredi MM Herl L Lee BM Spoelgen R Hshieh TT Ranganathan S Battey FD Liu CX Bacskai BJ Sever S Irizarry MC Strickland DK Hyman BT The low density lipoprotein receptor-related protein (LRP) is a novel beta-secretase (BACE1) substrate The Journal of biological chemistry 2005 280 17777 17785 10.1074/jbc.M414248200 15749709

[B22] Kim DY Ingano LA Carey BW Pettingell WH Kovacs DM Presenilin/gamma-secretase-mediated cleavage of the voltage-gated sodium channel beta2-subunit regulates cell adhesion and migration The Journal of biological chemistry 2005 280 23251 23261 10.1074/jbc.M412938200 15833746

[B23] Wong HK Sakurai T Oyama F Kaneko K Wada K Miyazaki H Kurosawa M De Strooper B Saftig P Nukina N beta Subunits of voltage-gated sodium channels are novel substrates of beta-site amyloid precursor protein-cleaving enzyme (BACE1) and gamma-secretase The Journal of biological chemistry 2005 280 23009 23017 10.1074/jbc.M414648200 15824102

[B24] Hu X Hicks CW He W Wong P Macklin WB Trapp BD Yan R Bace1 modulates myelination in the central and peripheral nervous system Nature neuroscience 2006 9 1520 1525 10.1038/nn1797 17099708

[B25] Willem M Garratt AN Novak B Citron M Kaufmann S Rittger A DeStrooper B Saftig P Birchmeier C Haass C Control of peripheral nerve myelination by the beta-secretase BACE1 Science 2006 314 664 666 10.1126/science.1132341 16990514

[B26] Hu X He W Diaconu C Tang X Kidd GJ Macklin WB Trapp BD Yan R Genetic deletion of BACE1 in mice affects remyelination of sciatic nerves Faseb J 2008 22 2970 2980 10.1096/fj.08-106666 18413858 PMC2493455

[B27] Cai H Wang Y McCarthy D Wen H Borchelt DR Price DL Wong PC BACE1 is the major beta-secretase for generation of Abeta peptides by neurons Nature Neurosci 2001 4 233 234 10.1038/85064 11224536

[B28] Roberds SL Anderson J Basi G Bienkowski MJ Branstetter DG Chen KS Freedman SB Frigon NL Games D Hu K BACE knockout mice are healthy despite lacking the primary beta-secretase activity in brain: implications for Alzheimer's disease therapeutics Hum Mol Genet 2001 10 1317 1324 10.1093/hmg/10.12.1317 11406613

[B29] Dominguez D Tournoy J Hartmann D Huth T Cryns K Deforce S Serneels L Camacho IE Marjaux E Craessaerts K Roebroek AJ Schwake M D'Hooge R Bach P Kalinke U Moechars D Alzheimer C Reiss K Saftig P De Strooper B Phenotypic and biochemical analyses of BACE1- and BACE2-deficient mice The Journal of biological chemistry 2005 280 30797 30806 10.1074/jbc.M505249200 15987683

[B30] Savonenko AV Melnikova T Laird FM Stewart KA Price DL Wong PC Alteration of BACE1-dependent NRG1/ErbB4 signaling and schizophrenia-like phenotypes in BACE1-null mice Proc Natl Acad Sci USA 2008 105 5585 5590 10.1073/pnas.0710373105 18385378 PMC2291091

[B31] Catterall WA From ionic currents to molecular mechanisms: the structure and function of voltage-gated sodium channels Neuron 2000 26 13 25 10.1016/S0896-6273(00)81133-2 10798388

[B32] Yu FH Westenbroek RE Silos-Santiago I McCormick KA Lawson D Ge P Ferriera H Lilly J DiStefano PS Catterall WA Scheuer T Curtis R Sodium channel beta4, a new disulfide-linked auxiliary subunit with similarity to beta2 J Neurosci 2003 23 7577 7585 12930796 10.1523/JNEUROSCI.23-20-07577.2003 PMC6740763

[B33] Isom LL Sodium channel beta subunits: anything but auxiliary Neuroscientist 2001 7 42 54 10.1177/107385840100700108 11486343

[B34] Lai HC Jan LY The distribution and targeting of neuronal voltage-gated ion channels Nat Rev Neurosci 2006 7 548 562 10.1038/nrn1938 16791144

[B35] Kim DY Carey BW Wang H Ingano LA Binshtok AM Wertz MH Pettingell WH He P Lee VM Woolf CJ Kovacs DM BACE1 regulates voltage-gated sodium channels and neuronal activity Nat Cell Biol 2007 9 755 764 10.1038/ncb1602 17576410 PMC2747787

[B36] Huth T Schmidt-Neuenfeldt K Rittger A Saftig P Reiss K Alzheimer C Non-proteolytic effect of beta-site APP-cleaving enzyme 1 (BACE1) on sodium channel function Neurobiology of disease 2009 33 282 289 10.1016/j.nbd.2008.10.015 19056495

[B37] Hitt B O'Connor T Maus E Vassar RJ BACE1 as a potential mediator of stress response in the brain 2008 Washington, DC: Society for Neuroscience Program No. 438.9. 2008 Neuroscience Meeting Planner, Online

[B38] Wallace RH Wang DW Singh R Scheffer IE George AL Phillips HA Saar K Reis A Johnson EW Sutherland GR Berkovic SF Mulley JC Febrile seizures and generalized epilepsy associated with a mutation in the Na+-channel beta1 subunit gene SCN1B Nat Genet 1998 19 366 370 10.1038/448 9697698

[B39] Claes L Del-Favero J Ceulemans B Lagae L Van Broeckhoven C De Jonghe P De novo mutations in the sodium-channel gene SCN1A cause severe myoclonic epilepsy of infancy Am J Hum Genet 2001 68 1327 1332 10.1086/320609 11359211 PMC1226119

[B40] Audenaert D Claes L Ceulemans B Lofgren A Van Broeckhoven C De Jonghe P A deletion in SCN1B is associated with febrile seizures and early-onset absence epilepsy Neurology 2003 61 854 856 14504340 10.1212/01.wnl.0000080362.55784.1c

[B41] Scheffer IE Harkin LA Grinton BE Dibbens LM Turner SJ Zielinski MA Xu R Jackson G Adams J Connellan M Petrou S Wellard RM Briellmann RS Wallace RH Mulley JC Berkovic SF Temporal lobe epilepsy and GEFS+ phenotypes associated with SCN1B mutations Brain 2007 130 100 109 10.1093/brain/awl272 17020904

[B42] Holland KD Kearney JA Glauser TA Buck G Keddache M Blankston JR Glaaser IW Kass RS Meisler MH Mutation of sodium channel SCN3A in a patient with cryptogenic pediatric partial epilepsy Neurosci Lett 2008 433 65 70 10.1016/j.neulet.2007.12.064 18242854 PMC2423278

[B43] Misra SN Kahlig KM George AL Impaired NaV1.2 function and reduced cell surface expression in benign familial neonatal-infantile seizures Epilepsia 2008 49 1535 1545 10.1111/j.1528-1167.2008.01619.x 18479388 PMC3647030

[B44] Meisler MH Kearney JA Sodium channel mutations in epilepsy and other neurological disorders J Clin Invest 2005 115 2010 2017 10.1172/JCI25466 16075041 PMC1180547

[B45] Burgess DL Noebels JL Single gene defects in mice: the role of voltage-dependent calcium channels in absence models Epilepsy Res 1999 36 111 122 10.1016/S0920-1211(99)00045-5 10515159

[B46] Chung WK Shin M Jaramillo TC Leibel RL LeDuc CA Fischer SG Tzilianos E Gheith AA Lewis AS Chetkovich DM Absence epilepsy in apathetic, a spontaneous mutant mouse lacking the h channel subunit, HCN2 Neurobiol Dis 2009 33 499 508 10.1016/j.nbd.2008.12.004 19150498 PMC2643333

[B47] Browne TR Browne TR, Feldman RG Ethosuximide (Zarontin) and other succinimides Epilepsy: Diagnosis and Management 1983 Boston, Mass: Little Brown & Co 215 224

[B48] Heller AH Dichter MA Sidman RL Anticonvulsant sensitivity of absence seizures in the tottering mutant mouse Epilepsia 1983 24 25 34 10.1111/j.1528-1157.1983.tb04862.x 6401628

[B49] Racine RJ Gartner JG Burnham WM Epileptiform activity and neural plasticity in limbic structures Brain Res 1972 47 262 268 10.1016/0006-8993(72)90268-5 4641271

[B50] Hu RQ Koh S Torgerson T Cole AJ Neuronal stress and injury in C57/BL mice after systemic kainic acid administration Brain Res 1998 810 229 240 10.1016/S0006-8993(98)00863-4 9813346

[B51] Zhao J Fu Y Yasvoina M Shao P Hitt B O'Connor T Logan S Maus E Citron M Berry R Binder L Vassar R Beta-site amyloid precursor protein cleaving enzyme 1 levels become elevated in neurons around amyloid plaques: implications for Alzheimer's disease pathogenesis J Neurosci 2007 27 3639 3649 10.1523/JNEUROSCI.4396-06.2007 17409228 PMC6672403

[B52] Hu X Zhou X He W Yang J Xiong W Wong P Wilson CG Yan R BACE1 deficiency causes altered neuronal activity and neurodegeneration J Neurosci 2010 30 8819 8829 10.1523/JNEUROSCI.1334-10.2010 20592204 PMC2902368

[B53] Huguenard JR McCormick DA Thalamic synchrony and dynamic regulation of global forebrain oscillations Trends Neurosci 2007 30 350 356 10.1016/j.tins.2007.05.007 17544519

[B54] Chiocco MJ Lamb BT Spatial and temporal control of age-related APP processing in genomic-based beta-secretase transgenic mice Neurobiol Aging 2007 28 75 84 10.1016/j.neurobiolaging.2005.11.011 16387391 PMC2659539

[B55] Cirrito JR Yamada KA Finn MB Sloviter RS Bales KR May PC Schoepp DD Paul SM Mennerick S Holtzman DM Synaptic activity regulates interstitial fluid amyloid-beta levels in vivo Neuron 2005 48 913 922 10.1016/j.neuron.2005.10.028 16364896

[B56] Kamenetz F Tomita T Hsieh H Seabrook G Borchelt D Iwatsubo T Sisodia S Malinow R APP processing and synaptic function Neuron 2003 37 925 937 10.1016/S0896-6273(03)00124-7 12670422

[B57] Hsieh H Boehm J Sato C Iwatsubo T Tomita T Sisodia S Malinow R AMPAR removal underlies Abeta-induced synaptic depression and dendritic spine loss Neuron 2006 52 831 843 10.1016/j.neuron.2006.10.035 17145504 PMC1850952

[B58] Cortez MA Wu Y Gibson KM Snead OC Absence seizures in succinic semialdehyde dehydrogenase deficient mice: a model of juvenile absence epilepsy Pharmacol Biochem Behav 2004 79 547 553 10.1016/j.pbb.2004.09.008 15582027

